# Burnout in Intensive Care Nurses during the COVID-19 Pandemic: A Scoping Review on Its Prevalence and Risk and Protective Factors

**DOI:** 10.3390/ijerph191912914

**Published:** 2022-10-09

**Authors:** Ferdinando Toscano, Francesco Tommasi, Davide Giusino

**Affiliations:** 1 Department of Psychology, University of Bologna, 40127 Bologna, Italy; 2 Department of Human Sciences, University of Verona, 37129 Verona, Italy

**Keywords:** COVID-19, SARS-CoV-2, burnout, nurses, intensive care units

## Abstract

Background: The COVID-19 pandemic has strained hospitals and healthcare workers engaged in combating the virus with limited knowledge and resources. Intensive care unit (ICU) nurses are among the healthcare workers most affected by the pandemic and are at risk for developing burnout syndrome. Objective: The present study aims to explore burnout symptoms prevalence among ICU nurses and to identify the individual, organizational, and contextual risk, and protective factors of burnout in ICU nurses during the COVID-19 pandemic. Methods: A scoping review was conducted by searching PubMed, Scopus, and Web of Science. Only papers with empirical data and referred to ICU nurses were included. A total of 350 initial results were yielded, and 40 full texts were screened. Twelve papers constituted the final sample in the analysis. Results: High levels of symptoms of burnout (emotional exhaustion, depersonalization, and reduced personal accomplishment) were registered among ICU nurses during the COVID-19 pandemic. Increased workload, lack of equipment, social stigma, and fear of contagion emerged as key risk factors. Social support from leaders and colleagues, professional recognition, use of personal protective tools, and witnessing patients’ successful recovery emerged as major protective factors. Conclusions: The results may inform the development of timely actions to counter burnout in ICU nurses during this COVID-19 pandemic and in a post-COVID-19 scenario.

## 1. Introduction

The COVID-19 (or SARS-CoV-2) pandemic has increased the workload of healthcare personnel [1] with the growth of workers’ physical, psychological, and technical efforts [2,3]. Since the pandemic outbreak, scholars have reported health workers’ experiences of psychological disorders such as increased irritability, anxiety, sleep disorders, muscle tension, nightmares, nervous breakdowns, and other negative psychophysical phenomena [4,5]. Furthermore, healthcare workers’ continuous exposure to emergencies represents a form of prolonged work-related stress, which leads healthcare workers to suffer physical, emotional, and mental fatigue, namely, Burn-Out Syndrome (BOS) [6,7].

BOS is work-related stress involving employees’ emotional exhaustion, depersonalization, and reduced personal accomplishment [8]. Emotional exhaustion is defined as workers feeling fatigued and unable to face the demands of their job or engage with people. Depersonalization is described as negative or inappropriate attitudes towards users, irritability, loss of idealism, and withdrawal. Reduced personal accomplishment is considered a reduction in productivity or ability, low morale, and an inability to cope [8]. In studies on nurses, BOS appears with frustration, anxiety, suicidal ideation [9,10], reduction in caregiving quality [11], decreased patient satisfaction [12], and increased risk of hospital infections [13].

Scholars from various disciplines have recently heightened their attention on BOS among nurses in the pandemic context [14,15,16]. However, studies explicitly encompassing BOS among intensive care unit (ICU) nurses remain sparse. To date, a few literature reviews on BOS prevalence during the COVID-19 pandemic in healthcare workers in multiple roles, not centered on ICU nurses, have been conducted [17,18,19,20]. Other contributions have focused on nurses working in more than one ward [21], or comparisons between healthcare workers with different roles but working in single-type wards [22]. Yet, the question about BOS prevalence among ICU nurses during the COVID-19 pandemic remains unanswered as no previous reviews have focused on it. Notably, no studies have explicitly looked for factors associated with BOS among ICU nurses during the pandemic. Nevertheless, among health care personnel, ICU nurses experienced the highest increase in workload during the pandemic [23], and at a time when work peaks—such as that for COVID-19—are not ruled out and when ICU work remains permeated by very high patient fragility, the identification of the psychosocial risks associated with this work is essential.

Since the pandemic spread, ICUs have been dealing with unexpected transformations. Changes in shifts, an augmented need for end-of-life management, increased use of full-body personal protective equipment (PPE), device-based communications with patients’ families, and lack of auxiliary staff to support nursing activities are among the most reported consequences of the onset of the pandemic scenario [24,25,26,27,28]. ICU nurses are in constant contact with COVID-19-positive patients, whereby the risks of this infectious disease entail [24]. However, early investigations on the relationships between such dynamics and BOS are sparse [6,7]. The knowledge of the individual, organizational, and contextual factors underpinning BOS prevalence is limited, and questions on the BOS prevalence among ICU nurses during the COVID-19 pandemic remain unanswered. Given the centrality of ICU nurses’ work during the pandemic, there is a pressing need to understand this phenomenon better.

This article aims to present the findings from a scoping review of the empirical literature regarding BOS among ICU nurses during the COVID-19 pandemic. The main objective was to gather state-of-the-art data about the prevalence of the individual, organizational, and contextual factors associated with BOS among ICU nurses during the pandemic. The prevalence of BOS among ICU nurses is a well-established topic in the literature, as ICU nurses work in wards where patients are in precarious conditions and need extensive care [29]. Since the spread of the COVID-19 pandemic, it appears to be necessary to investigate the risk of BOS and the individual, organizational, and contextual factors associated with it to guide the practice of this profession in the present and future. The rationale behind a scoping synthesis of the currently available empirical data lies in the awareness of the prolonged pressure and stress on the ICU facility during COVID-19 [30]. In this vein, the broad objective of the present paper is to address the following two questions:

- How prevalent are BOS symptoms among ICU nurses during the COVID-19 pandemic?
- Which individual, organizational, and contextual factors are associated with the risk of, and protection against, BOS among ICU nurses during the COVID-19 pandemic?

The following sections introduce the methods we deployed to search and extract sources related to our research aims. Next, we report our results, which are synthesized and discussed in the last section. Finally, from a psychological and occupational approach, we offer research insights and practical implications of our findings to guide researchers and practitioners toward solutions that impact the health and quality of the working life of critical care nurses.

## 2. Methods

### 2.1. Study Design

Based on the methodological indications by Arksey and O’Malley [31], we conducted a scoping review to synthesize the empirical literature about BOS among ICU nurses since the COVID-19 pandemic outbreak. Unlike systematic reviews, which aim to assess and summarize the evidence-base around specific topics, a scoping review aims to capture the evidence’s breadths, and map, condense, and identify the known and the unknown in a particular content area of the literature. Scoping reviews are nevertheless based on a systematic approach to data collection. Yet, eligibility criteria for inclusion and exclusion allow more flexibility, and the synthesis is based on the research questions rather than methodological rigor and strict assessment. Considering the current lack of comprehensive knowledge about BOS among ICU nurses during the COVID-19 pandemic and the pressing need for providing such knowledge, we deemed the scoping review method suitable for the present study’s aims.

### 2.2. Keywords and Search Query

We anchored our literature search on the research questions. Therefore, the whole research team first broke down and refined the research questions to identify the best keywords the final search string would include. After a pilot search, the final search string with Boolean operators was as follows: “nurs*” AND (“burnout” OR “burn-out”) AND (“COVID-19” OR “SARS-CoV-2”) AND (“intensive care” OR “ICU” OR “critical care” OR “intensive therapy” OR “acute care” OR “intensive-care”).

### 2.3. Bibliographical Databases

We undertook the structured search on three data sources: PubMed, Scopus, and Web of Science (WoS). We selected these bibliographical databases to ensure good multi-disciplinary coverage of high-quality peer-reviewed articles [32]. We limited the search-time range from November 2019 (i.e., pandemic outbreak) until the search date, 9 September 2022. No restrictions were set for language, study type, and publication stage, as we wanted to ensure that no relevant contributions could be missed.

### 2.4. Inclusion and Exclusion Criteria

We evaluated the collected data against the agreed inclusion and exclusion criteria. The inclusion criteria we used to select the studies for the review were the following: (a) studies with only nurses as participants (or with participants with other roles present for less than 3% of the sample); (b) studies with participants working only in ICUs, so that the experience of nurses working only in these units could be delineated; (c) studies in which the BOS experienced during the COVID-19 pandemic was explicitly mentioned; (d) studies with empirical data, with a quantitative, qualitative, or mixed research design. As for exclusion criteria, we agreed to exclude: (a) studies with participants who were not nurses or not only nurses (excluding a 3% tolerance, e.g., including doctors, auxiliary staff, administrative staff, etc.); (b) studies that considered the experience of nurses working in wards other than intensive care, or comparable units, only; (c) studies that never mentioned BOS during the COVID-19 pandemic, though also concerning similar concepts; (d) studies with a design other than quantitative, qualitative, or mixed, e.g., opinions/editorials, since they were not peer-reviewed and/or did not report empirical data.

Two study researchers worked together to select papers to be included in the review. First, we screened titles to remove duplicate articles (i.e., same articles found across different bibliographical databases). Then, we screened abstracts to remove studies that did not meet one or more inclusion criteria. Finally, we read the remaining full-text papers to detail the results.

### 2.5. Methodological Quality Assessment

We assessed the methodological quality of the selected studies using the JBI Critical Appraisal Tools. In particular, the 8-item JBI Critical Appraisal Checklist for Analytical Cross-Sectional Studies [33] (see Appendix A) and the 10-item JBI Critical Appraisal Checklist for Qualitative Research [34] (see Appendix B) were used. These methods allow for the critique or appraisal of collected research evidence to determine the extent to which a study has addressed the possibility of bias in its design, conduction, and analysis. Items in JBI Critical Appraisal Checklist for Analytical Cross-Sectional Studies encompass criteria for inclusion in the sample, detailed description of the study subjects and setting, valid and reliable measurements, management of confounding factors, and the use of appropriate statistical analyses. Items in JBI Critical Appraisal Checklist for Qualitative Research encompass congruity that the research methodology has with the adopted philosophical perspective, the research questions or objectives, the deployed data collection methods, the representation and analysis of data, their interpretation, cultural or theoretical location of the researcher(s), the representation of participants’ voices, and the ethical aspects. This process was conducted by the same two researchers engaged in the previous step.

### 2.6. Data Extraction

We extracted evidence from the gathered studies as a last step of the search process. We extracted data from studies through a structured table, built ad hoc for this study. It reports, for each study, the authors and year, scientific journal, country where the study was conducted, data collection period, main objectives, number of participants, research design, measures, and the main results. In this case, the two researchers engaged in the previous steps were responsible for the first draft of the table. The third study researcher reviewed the previous extraction work.

### 2.7. Ethical Considerations

This study gathers results from other studies and fully complies with the Helsinki declaration on medical research [35]. Since it does not involve research participants, no ethical approval was required.

## 3. Results

**Figure 1:**
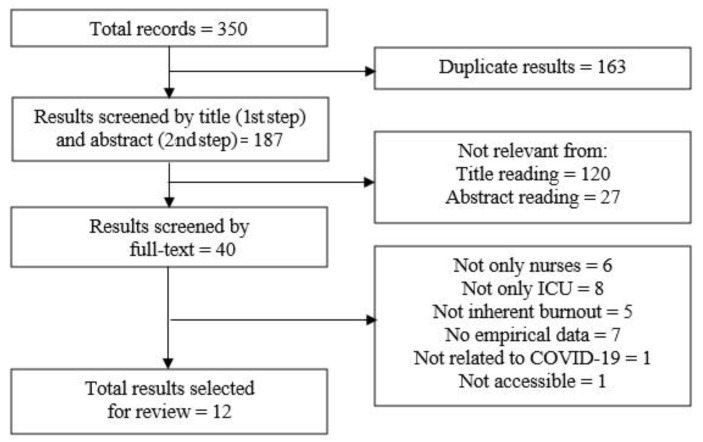
Process and results of the search strategy deployed in the current study.

Figure 1 shows the process and results of our search strategy. The search yielded an initial *N* = 350 (i.e., *n* = 127 on PubMed, *n* = 116 on Scopus, *n* = 107 on WoS). Of these, 163 were excluded after title screening as they were duplicate items. Then, the titles and abstracts of the remaining 187 items were screened, and 147 were excluded as they met the exclusion criteria. Finally, the full texts of the remaining 40 items were screened, and 28 were excluded as they did not meet the inclusion criteria. This process left a final sample composed of 12 eligible studies included in our review for further analysis.

### 3.1. Methodological Quality Assessment of the Selected Studies

Table 1 and Table 2 show how we applied the JBI Critical Appraisal Tools to conduct the methodological quality assessment of the twelve selected studies. Table 1 was applied to all quantitative studies and to the quantitative component of the mixed-method studies. Table 2 was applied to all qualitative studies and to the qualitative component of the mixed-method studies. The assessment instrument items are reported in Appendix A and Appendix B. Each study was characterized by specific peculiarities, allowing for a diverse evaluation of their methodological quality. Furthermore, the twelve studies come from different journals and publishers, suggesting the absence of common-source bias.

### 3.2. Overview of the Selected Studies

Six studies [36,37,41,42,43,44] deployed survey-based quantitative methods with psychometric measurements and three of them [37,43,44] administered Maslach and Jackson’s [48] instrument to directly measure the three components of participants’ BOS, such as emotional exhaustion, depersonalization, and reduced personal accomplishment. Three studies [45,46,47] have a qualitative, individual interview-based design. Three other studies [38,39,40] are characterized by the use of mixed methods, that is, two of them [38,39] combined the administration of psychometric scales with written open-ended questions, which constitute a qualitative data collection and focus more specifically on ICU nurses’ burnout and related issues, whereas another one [40] deployed focus groups. Three studies took place in the United States [39,45,47], two in Turkey [41,46], one in Sweden [36], one in Belgium [37], one in Canada [38], one in Israel [40], one in South Africa [42], one in Iran [43], and one in Italy [44]. The main characteristics of the reviewed studies are summarized in Table 3.

### 3.3. Prevalence of BOS among ICU Nurses during the COVID-19 Pandemic

Regarding BOS prevalence among ICU nurses during the COVID-19 pandemic, seven studies [37,38,40,41,42,43,44] provided evidence of the presence of BOS symptoms in the ICU nurses participating in the studies, thus, supporting answers to the first research question of the present review. In the Belgian context [37], 68% out of the 1100 participating ICU nurses showed BOS symptoms. Thirty-eight percent reported emotional exhaustion (EE) values above the threshold, 29% were at risk of developing depersonalization (DP) symptoms, and 31% showed reduced personal accomplishment (PA). In a Canadian sample composed of 425 ICU nurses, Crowe et al. [38] found moderate-to-high burnout in all (100%) nurses, with 87% suffering from signs of secondary traumatic stress and 22% intended to quit their current employment. In Israel, Kagan et al. [40] found that, in a convenience sample comprising 100 registered nurses working in the ICU division, nurses reported high levels of burnout (M = 3.44, SD = 1.37, 1–7 scale), with 66% of the nurses reporting varying levels of involvement in the direct treatment of COVID-19 patients and burnout, contributing significantly to the variance of the professional functioning among nurses caring for COVID-19 patients. In a Turkish sample composed of 116 intensive care nurses, Kurt Alkan et al. [41] found a mean total score of burnout of 50.75 out of 70, as well as a significant positive correlation between nurses’ burnout and fear of COVID-19. As shown by Ndlovu et al. [42], the majority of the 225 involved South African nurses working in critical care units during the first wave of COVID-19 experienced moderate-to-high burnout, that is, 26.6% scored below 43 (low burnout), 46.1% scored between 43 and 56 (moderate burnout), and 27.3% scored higher than 56 (high burnout). Among 140 nurses working in Iranian ICUs, Omidi et al. [43] found that 45% of them showed moderate-to-high emotional exhaustion, 43.6% showed moderate-to-high depersonalization, and 5% reported low personal accomplishment. Additionally, this study highlighted a negative association between emotional exhaustion, depersonalization of burnout, and quality of life (r = −0.47 to −0.79). Finally, an Italian sample [44] composed of 291 participants returned values above the threshold for all three components of BOS, such as EE for 90.4%, DP for 8.2%, and reduced PA for 24.4% of respondents. Interestingly, almost half of the participants in this study were not working in the ICU before the pandemic outbreak.

Despite some variability in the levels of the three components of BOS, the reviewed studies support the statement that ICU nurses during the COVID-19 pandemic were at relevant risk of developing BOS.

### 3.4. Individual, Organizational, and Contextual Factors Related to BOS among ICU Nurses during the COVID-19 Pandemic

#### 3.4.1. Results from Quantitative Studies

Regarding the factors underlying BOS prevalence among ICU nurses during the COVID-19 pandemic, the selected quantitative studies provided an additional understanding of the individual- and organizational-level factors associated with BOS (see also Table 4). At the individual level, factors such as gender were related to BOS. Vitale et al. [44] found that female ICU nurses had higher levels of emotional exhaustion than male ICU nurses. At the organizational level, Bergman et al. [36] found that the choice of health care institutions to place nurses in intensive care units who did not specialize in this type of care, combined with the high workload, generated a feeling among study participants that they were not always able to provide adequate nursing care. This resulted in ethical stress, which has been linked to burnout. Additionally, Bruyneel et al. [37] identified factors that were correlated with increased BOS risks, such as a higher number of patients to care for compared to before the COVID-19 pandemic outbreak, high workload, lack of PPE, and unavailability of tests to assess whether they had been infected with the virus. Finally, Ndlovu et al. [42] highlighted that the high workload, which may have been associated with the COVID-19 pandemic, negatively influenced nurses’ professional quality of life, which can be a result of burnout itself.

#### 3.4.2. Results from Qualitative and Mixed-Method Studies

The identification of the individual-, organizational-, and contextual-level factors associated with the risk of developing BOS was more extensively addressed in the qualitative [45,46,47] and mixed-methods [38,39,40] studies selected for the present review. Cadge et al. [45] and Crowe et al. [38] emphasized relationships with colleagues and leadership support, especially, as key organizational factors facilitating individual and group well-being and minimizing BOS risks. In this sense, the authors refer to new work challenges that COVID-19 brought, including the need to re-arrange workgroups (often by moving people from other departments), re-discuss existing roles, or tolerate more uncertain and less-defined working conditions. These endeavors required support from colleagues but even greater efforts from the nursing leadership in providing information, maintaining morale, and redesigning work procedures to not negatively affect the workers’ well-being. Similarly, Guttormson et al. [39] reported that, beyond increased workload, the rawness of taking care of ICU patients during the pandemic represented one of the main organizational factors contributing to BOS. Seeing patients dying while being far from their loved ones, feeling helpless in front of the pandemic situation, reflecting on the fact that these people died despite being mentally lucid—witnessing such a scenario daily was reported as highly related to BOS, especially in terms of emotional exhaustion and reduced personal accomplishment. Additionally, Çelik and Kiliç [46] reported that nurses suffered from family relationship breakdown and insufficiency in intrafamilial coping, and reported to live a tiring life with great responsibility and face mental problems, including burnout syndrome. Moreover, although such stark realities have occurred over the past two years in ICUs due to COVID-19, the studies [39,45] also point out how exposure of ICU nurses to contextual, societal, and institutional factors may contribute to BOS risk. This is the case with the rejection of COVID-19 or the lack of recognition of health workers by segments of society. In this sense, some people’s distrust of ICU nurses, lack of attention to their psychological health by healthcare agencies, and fear of infecting family and friends represented harmful contextual conditions that increased the pressure on ICU nurses’ mental health during the COVID-19 pandemic. As a result of a one-to-one interview study asking nurses general questions about their lived experiences of the pandemic, Christianson et al. [47] reported that nurses felt betrayal at perceived breeches in their duty-of-care agreement by their employers, the general public, and national health authorities. They experienced alterations to previous standards of care such as significantly increased workload, worsening understaffing, and changes to patient-care expectations that were implemented for reasons not concerning the betterment of patient care. Nurses reported to feel a moral obligation to provide care while experiencing disempowerment and burnout, which affected them both in and out of the workplace. Similar themes were referred to by Crowe et al. [38], such as a sense of disillusionment, defeat, and intent to leave.

Although COVID-19 constitutes an unpleasant and harmful challenge to the world, Guttormson et al. [39] and Kagan et al. [40] also suggest some positive aspects reported by ICU nurses which may prevent the risk of developing BOS. Among these, ICU nurses mentioned new work challenges, the healing of some patients, the appreciation by them, their families and the community, and the recognition of ICU nurses as figures collectively acquiring salience. We refer to Table 4 for further details on the results of the reviewed studies.

**Table 4:** Main results of the reviewed studies.

Reference	Study Period	Participants	Measures	Main Results
Bergman et al., 2021 [36]	May 2020	151 ICU nurses (131 moved to the ICU due to pandemic)	Thirteen multiple-choice questions, including questions about participants’ specialist training, years of clinicalexperience, workplace, number of patients per shift, and introductionand training regarding COVID-19 patients.	The situation of not being able to provide nursing care resulted in ethical stress and led to an increased workload and worsened work environment, which affected nurses’ health and well-being.
Bruyneel et al., 2021 [37]	April–May 2020	1135 ICU nurses	Maslach Burnout Inventory (MBI)	Overall, 68% of ICU nurses were at risk of BOS. A total of 29% were at risk for depersonalization (DP), 31% for reduced personal accomplishment (PA), and 38% for emotional exhaustion (EE). A nurse–patient ratio of 1:3 increased the risk of EE and DP. High workload was associated with a higher risk for BOS. A lack of protective equipment increased the risk of EE. The presence of COVID-19-like symptoms without being tested increased the risk of EE.
Cadge et al., 2021 [45]	June–August 2020	16 ICU nurses (8 already in ICU, 8 moved to ICU due to pandemic)	Semi-structured interviews with questions about working under unfamiliar circumstances, caring for patients with a new infectious disease, risks to themselves and their family, ideas on additional support they would find helpful.	Participants emphasized the importance of nurse leadership support during this experience. Leadership practices that maximize visibility and support facilitated individual and group well-being and minimized BOS risk.
Çelik and Kiliç, 2022 [46]	May–June 2020	18 ICU nurses	Individual in-depth interview: two questions were asked by the authors, namely: (1) Can you explain the effects of the COVID-19 pandemic on your relations with your family? (2) How does the COVID-19 pandemic affect you?	The study investigated three themes: breakdown in continuity of intrafamilial relationship, ineffectiveness in role performance, and ineffective individual coping. Nurses suffered from family relationship breakdown and insufficiency in intrafamilial coping; they reported to live a tiring life with great responsibility and face mental problems such as burnout syndrome and depression.
Christianson et al., 2022 [47]	November 2020–January 2021	13 ICU Nurses	One-on-one semi-structured interviews conducted by nurses following broad questions about the lived experience of nurses about the pandemic, e.g., can you tell me about what it has been like towork in the ICU during the COVID-19 pandemic?	Nurses reported betrayal at perceived breeches in their duty-of-care agreement by their employers, the society, and national health authorities. Alterations to previous standards of care such as significantly increased workloads, worsening understaffing, and changes to patient-care expectations that were implemented for reasons other than the betterment of patient care. Nurses reported to feel a moral obligation to provide care while experiencing disempowerment and burnout that affected them both in and out of the workplace.
Crowe et al., 2022 [38]	May–June 2021	425 ICU Nurses	Quantitative measures: the Impact of Event Scale—Revised (IES–R), the Depression, Anxiety, Stress Scale (DASS–21), the Professional Quality of Life scale (ProQoL), and the Intent to Turnover scale; Qualitative questions: optional open-ended question asking participants if there was anything else they wanted to share.	Nurses had symptoms of post-traumatic stress disorder (74%), depression (70%), anxiety (57%), and stress (61%). All (100%) nurses showed moderate-to-high burnout, 87% suffering from signs of secondary traumatic stress, and 22% intended to quit their current employment. Qualitative analysis of written comments submitted by 147 (34.5%) of the respondents depicted an immense mental health toll on the ICU that stemmed from (1) failed leadership and (2) the traumatic nature of the work environment, which led to (3) a sense of disillusionment, defeat, and an intent to leave.
Guttormson et al., 2022 [39]	October 2020–January 2021	285 ICU nurses	Closed questions on respondents’ characteristics, work setting, and challenges during the pandemic. Three open questions: (1) What do you want people to know about your experience during the COVID-19 pandemic? (2) Please, describe the greatest challenges you faced caring for COVID-19 patients. (3) Please, describe any positive things you observed or experienced during COVID-19.	Nurses reported stress due to a lack of evidence-based treatment, poor patient prognosis, and lack of family presence in the ICU. They perceived inadequate leadership support and inequity within working teams. They felt isolated due to a lack of consistent community efforts to slow the virus spread. Nurses reported exhaustion, anxiety, sleeplessness, moral distress, and fear of contracting COVID-19 or infecting family and friends.
Kagan et al., 2022 [40]	February–May 2021	115 ICU nurses (15 for the focus group and 100 for the cross-sectional study)	Quantitative measures: professional functioning, emotional stress at work, State Hope Scale, Nurses’ uncertainty, and Shirom–Melamed Burnout Measure; Qualitative measure: 15 focus groups.	Qualitative data analysis revealed challenges of the COVID-19 pandemic and positive aspects of the COVID-19 pandemic. Nurses reported high levels of burnout, emotional stress, and uncertainty, but moderate State Hope Scale scores, and moderate levels of professional functioning. State Hope Scale levels, uncertainty, and burnout variables contributed significantly and explained 46% of the variance of the professional functioning.
Kurt Alkan et al., 2022 [41]	February–April 2021	116 ICU nurses	Descriptive Information Form, COVID-19 Fear Scale, Depression, Anxiety and Stress Scale Short Form and Burnout Short Version, COVID-19 Fear Scale.	Strong associations among the presence of moderate-to-high symptoms of depression, anxiety, stress, and burnout levels among ICU nurses.
Ndlovu et al., 2022 [42]	January–May 2020	154 ICU nurses	Professional Quality of Life comprising dimensions of compassion satisfaction, burnout, and secondary traumatic stress disorder.	The high workload, which may have been associated with the COVID-19 pandemic, influenced nurses’ professional quality of life.
Omidi et al., 2022 [43]	July 2020–January 2021	140 ICU nurses	Maslach Burnout Inventory (MBI) and the WHO Quality of Life-BREF.	Positive associations between personal accomplishment and all dimensions of QoL and a negative association between emotional exhaustion, depersonalization of burnout and QOL dimensions.
Vitale et al., 2020 [44]	March–April 2020	291 ICU nurses (132 moved to ICU due to pandemic)	Maslach Burnout Inventory (MBI).	A total of 90.4% of the nurses reported above-threshold values for EE, 8.2% for DP, and 24.4% for reduced PA. Female nurses reported higher negative values than men for the only EE dimension.

## 4. Discussion

Following a call for a deeper understanding of ICU nurses’ work experience during the COVID-19 pandemic [45,49,50], the aim of this scoping review was twofold. First, it aimed to gather evidence about the prevalence of burnout syndrome among nurses in intensive care units during the COVID-19 pandemic. Second, it aimed to identify the individual-, organizational-, and contextual-level factors associated with the risk of and protection against BOS among ICU nurses during the COVID-19 pandemic, to comprehend and guide the present and the future. The picture emerging from the conducted analysis explains the complexity of studying ICU nurses’ BOS. Yet, it is possible to make preliminary considerations to guide the future for theory-building, conducting research studies, and implementing interventions.

Our review adopted strict inclusion criteria, substantially related to the need to extract in detail, and exclusively, the experience of ICU nurses grappling with patients with COVID-19. We found twelve studies matching our inclusion criteria, analyzing data collected from the outbreak of the pandemic to about the middle of 2021.

From the quantitative studies, including direct measures of BOS, it was not possible to derive numerical values that could generalize the prevalence of BOS in the ICU nurse population during the COVID-19 pandemic. This partly limits the possibility of answering the first objective of this study, related to the prevalence of BOS symptoms in ICU nurses during the pandemic. In addition, it complicates the possibility of making evaluations with available pre-pandemic data, if comparisons between periods were deemed necessary. In any case, the presence of BOS symptoms has proven to be a widespread phenomenon often involving more than half of the ICU nurses participating in the studies; this figure in itself is indicative of the significance of the phenomenon in the period and population studied.

The analyzed studies contributed to an emerging picture of the separate dimensions underlying BOS risks among ICU nurses during the COVID-19 pandemic. For instance, we found that low experience in ICU, high workload, unavailability of material (e.g., safety devices, personal protective equipment, etc.), low psychological support (e.g., lack of support from colleagues or the hospital structures), and social stigma are predisposing factors to the development of BOS in ICU nurses. Furthermore, our review reveals the presence of possible protective factors against BOS. These include witnessing patients’ successful recovery, support from colleagues, sympathetic nursing leadership, using individual protection tools, the appreciation of patients and their families, and the increasing recognition of ICU nurses’ role as a relevant professional category.

Although the COVID-19 pandemic is a complex phenomenon, the incidence of which varies in time and place, we believe that the knowledge of both risk and protective factors is a valuable resource, and can be adopted not only to cope with the still active COVID-19 pandemic, but also future potential other periods that may increase the demand for ICU care. Moreover, we consider the prevalence of BOS not only as a simple symptom related to moral distress and COVID-19-related exhaustion, but as a general condition related to uncertainty and prolonged exposure to continuous changes and work demands of ICU nurses, of which COVID-19 is a part but not the whole.

The presence of many extra-individual factors related to the presence of burnout symptoms corroborates this assumption. Organizational-, and contextual-level factors were reported as playing a relevant role in BOS development. These regarded working conditions related to the increase in job tasks and the organizational environment, with a relevant role played by the teamwork and the leadership, directly affecting ICU nurses’ morale. In addition, the overall ICU nurses’ experience may result in detrimental states, such as BOS, when social and contextual factors imbue a sense of loneliness and frustration, e.g., the lack of social recognition of healthcare professionals’ work. These findings strengthen a view of BOS resulting from a generally unpleasant and challenging state associated with the significance of ICU nurses’ work [39]. We, therefore, hope that, even now that COVID-19 seems to be becoming progressively less salient, national health systems will continue to focus their attention on ICU nurses, and offer them adequate resources so that the issues that COVID-19 has often exacerbated but almost never created can find the interest and commitment of health managers and, especially in public systems, policy makers.

### 4.1. Limitations and Recommendations for Future Research

Our scoping review has several limitations, which offer insights to future research to address the BOS issue among ICU nurses during the COVID-19 pandemic. While the specific nature of the study made it possible to investigate the phenomenon in the targeted occupational population, it must be acknowledged that the criteria chosen for this study did not allow for the integration of further research that, although investigating similar contexts, did not meet the strict conditions chosen for this study. However, the study provides a reasonable basis for investigating the BOS phenomenon among ICU staff grappling with COVID-19 and addressing the needs that emerged from them through the review of studies that concerned their experiences.

For theory-building and conduction of research, our study and its limitations can be considered to realize more “inclusive” studies in terms of search criteria, selection of articles, and variety of pandemic waves integrated by the considered scientific contributions. Although the COVID-19 pandemic is not yet over, it may affect intensive care units’ health delivery differently than in the past. One avenue of study is represented by the possibility of investigating these processes and dynamics, keeping into account the different timeframes of the pandemic. The incidence of BOS in intensive care units may have changed, and good practices to prevent it described in the literature would deserve significant research and dissemination attention.

In this vein, further analysis of the literature may include studies encompassing similar healthcare figures, for instance, nurses from emergency departments, which also faced increased workload and the presence of life-risk patients during the pandemic. Likewise, it has been reported that some ICU nurses operating in these departments during the pandemic did not originally belong to them but were used to operating in other hospital departments. Exploring potential differences between expert and novice ICU nurses using quantitative and qualitative methods would be interesting. While there may be similarities between ICU and emergency nurses, there might not be such similarities, for instance, with nurses from geriatric or orthopedic departments.

Finally, we believe it is necessary to further investigate the role of variables such as the number of patients served, increased work demands, teamwork, and how leadership influences nurses’ subjective experiences generally. Taking for granted the knowledge related to their role in the development of BOS symptoms in ICU nurses, we believe that future studies, of a more applied nature, should develop solutions for each of the macro-factors noted both at the individual level (as nurses develop BOS) and at the institutional level (challenging health systems). Therefore, we consider these elements crucial for investigating nurses’ experiences during a pandemic.

### 4.2. Practical Implications

Albeit this study does not provide definitive knowledge about BOS in ICU nurses during the COVID-19 pandemic, it holds some initial implications for practice. Given the evidence on the detrimental effects of BOS at the individual and institutional level, there is merit in considering strategies and training at the individual level to reduce the risk of BOS. From a multi-disciplinary perspective, healthcare professionals such as psychologists with interventions specific to this profession (e.g., mindfulness) would help ICU nurses better manage work-related stress.

Recognizing, however, the frequent impossibility of including dedicated figures devoted to taking care of ICU nurses’ well-being on the ward, we also consider peer-defusing training as an effective solution to create a space for group dialogue and verbalization of ICU nurses’ experiences [51]. Defusing works with groups of trained nurses reflecting on their working experience and conditions after their shift to reduce emotional fatigue and its accumulation during workweeks. This instrument may be helpful because it is challenging to provide expert psychological support, especially in facilities with a shortage of specific professionals consistently addressing the mental well-being of healthcare workers, or for cost reasons.

The reviewed studies also highlight the need for interventions to prevent BOS in ICU nurses, which can be achieved by adding support resources to nurses or eliminating the work limitations they report. In this sense, the improvement of the work equipment, the reduction in the load per shift, and better communication appear to be privileged intervention measures, not only to reduce the negative symptoms of burnout, but also to increase the personal accomplishment of these workers who, with better instruments, can develop a greater sense of effectiveness at work. Overall, the findings regarding individual, organizational, and contextual factors of BOS in critical care nurses could inform the design, development, and implementation of multi-level interventions to reduce BOS in health care workers. As known from the literature on interventions for promoting mental health at work [52,53,54], multi-level interventions are expected to be more effective than “single-level” interventions and, therefore, represent a prime avenue for addressing the BOS issue among ICU nurses.

## 5. Conclusions

The results from this scoping review point out that BOS is a concrete concern in the ICU nurses’ population during the COVID-19 pandemic, although substantial differences can be observed depending on local and temporal contexts. This research lists potential factors contributing to the increase or decrease in BOS risk among ICU nurses. These constitute good starting points for planning interventions to reduce BOS in ICU staff.

We believe the originality of the present paper rests in being one of the first syntheses of the literature on BOS exclusively among ICU nurses during the COVID-19 pandemic. We hope that the insights from this study will provide fertile ground for the development of interventions to address BOS in ICU nurses, considering that this issue does not just associate with the COVID-19 pandemic [55]. We hope, thus, to have provided food for thought not only for this period, but also for the post-pandemic period, to be used in normal times and even more if, unfortunately, other events should in the future arouse a greater need for ICU care in the world.

## Figures and Tables

**Table 1:** Methodological quality assessment of the reviewed studies (quantitative studies/parts).

	Bergman et al., 2021 [36]	Bruyneel et al., 2021 [37]	Crowe et al., 2022 [38]	Guttormson et al., 2022 [39]	Kagan et al., 2022 [40]	Kurt Alkan et al., 2022 [41]	Ndlovu et al., 2022 [42]	Omidi et al., 2022 [43]	Vitale et al., 2020 [44]
Q1	YES	YES	UNCLEAR	YES	YES	YES	UNCLEAR	YES	YES
Q2	YES	YES	YES	YES	YES	YES	YES	YES	YES
Q3	YES	YES	YES	UNCLEAR	YES	YES	YES	YES	UNCLEAR
Q4	YES	YES	YES	UNCLEAR	YES	YES	UNCLEAR	YES	UNCLEAR
Q5	YES	YES	YES	UNCLEAR	YES	YES	NO	YES	YES
Q6	YES	YES	YES	UNCLEAR	YES	YES	NO	YES	YES
Q7	YES	YES	YES	UNCLEAR	YES	YES	YES	YES	YES
Q8	YES	YES	YES	YES	YES	YES	YES	YES	YES

Note. Questions of the JBI Critical Appraisal Checklist for Analytical Cross-Sectional Studies available in Appendix A. N/A = Not applicable.

**Table 2:** Methodological quality assessment of the reviewed studies (qualitative studies/parts).

	Cadge et al., 2021 [45]	Çelik and Kiliç, 2022 [46]	Christianson et al., 2022 [47]	Crowe et al., 2022 [38]	Guttormson et al., 2022 [39]	Kagan et al., 2022 [40]
Q1	UNCLEAR	YES	YES	YES	YES	YES
Q2	YES	YES	YES	YES	YES	YES
Q3	YES	YES	YES	YES	YES	YES
Q4	YES	YES	YES	YES	YES	YES
Q5	YES	YES	YES	YES	YES	YES
Q6	YES	YES	YES	YES	YES	YES
Q7	YES	YES	YES	YES	UNCLEAR	YES
Q8	YES	YES	YES	YES	YES	YES
Q9	YES	YES	YES	YES	YES	YES
Q10	YES	YES	YES	YES	YES	YES

Note. Questions of the JBI Critical Appraisal Checklist for Qualitative Research available in Appendix B. N/A = Not applicable.

**Table 3:** Main characteristics of the reviewed studies.

Reference	Scientific Journal	Publisher	Country	Study Period	Participants	Method	Main Goals
Bergman et al., 2021 [36]	Nursing in Critical Care	Wiley	Sweden	May 2020	151 ICU nurses (131 moved to ICU due to pandemic)	Quantitative	Describe ICU nurses’ experiences of caring for patients with COVID-19 in ICUs during the pandemic.
Bruyneel et al., 2021 [37]	Intensive and Critical Care Nursing	Elsevier	Belgium	April–May 2020	1135 ICU nurses	Quantitative	Assess the BOS risk prevalence and identify risk factors among ICU nurses during the COVID-19 pandemic.
Cadge et al., 2021 [45]	Journal of Nursing Management	Wiley	United States	June– August 2020	16 ICU nurses (8 already in ICU, 8 moved to ICU due to pandemic)	Qualitative	Understand how nurses experienced care of COVID-19-positive patients within ICUs.
Çelik and Kiliç, 2022 [46]	World Journal of Clinical Cases	Baishideng Publishing Group	Turkey	May–June 2020	18 ICU nurses	Qualitative	Explore nurses’ anxiousness about themselves, their children and family, and inability to cope with the situation during the pandemic.
Christianson et al., 2022 [47]	SAGE Open Nursing	Sage	United States	November 2020–January 2021	13 ICU Nurses	Qualitative	Examine the impact of the COVID-19 pandemic on the duty-of-care balanceamong ICU nurses who manage COVID-19 patients.
Crowe et al., 2022 [38]	Intensiveand Critical Care Nursing	Springer	Canada	May–June 2021	425 ICU Nurses	Mixed methods	Examine the impact of the COVID-19 pandemic on ICU nurses’ mental health, quality of work life, and intent to stay in their current positions.
Guttormson et al., 2022 [39]	American Journal of Critical Care	American Association of Critical-Care Nurses	United States	October 2020–January 2021	285 ICU nurses	Mixed methods	Describe the experiences of US ICU nurses in the COVID-19 pandemic.
Kagan et al., 2022 [40]	Journal of Nursing Scholarship	Wiley	Israel	February–May 2021	115 ICU nurses (15 for the focus group and 100 for the cross-sectional study)	Mixed methods	Examine the challenges of operating and managing intensive care units during the COVID-19 pandemic among ICU nurse managers, and the relationships between uncertainty, stress, burnout, hope, and professional functioning among intensive care nurses during the COVID-19 pandemic.
Kurt Alkan et al., 2022 [41]	OMEGA—Journal of Death and Dying	Sage	Turkey	February–April 2021	116 ICU nurses	Quantitative	Examine the relation between the fear of COVID-19 and depression, anxiety, and burnout of ICU nurses during the pandemic.
Ndlovu et al., 2022 [42]	Southern African Journal of Critical Care	Critical Care Society of Southern Africa	South Africa	January–May 2020	154 ICU nurses	Quantitative	Describe the demographic factors associated with professional quality of life of critical care nurses working in Gauteng, South Africa.
Omidi et al., 2022 [43]	Journal of Neonatal Nursing	Springer	Iran	July 2020–January 2021	140 ICU nurses	Quantitative	Determine the association between burnout and nurses’ quality of life in ICU nurses during the pandemic.
Vitale et al., 2020 [44]	Minerva Psichiatrica	Minerva Medica	Italy	March–April 2020	291 ICU nurses (132 moved to ICU due to pandemic)	Quantitative	Assess the BOS level among ICUs nurses caring for COVID-19-positive Patients.

## Data Availability

Not applicable.

## Appendix A

**Table A1:** JBI Critical Appraisal Checklist for Analytical Cross-Sectional Studies.

	Yes	No	Unclear	Not Applicable
Were the criteria for inclusion in the sample clearly defined?	□	□	□	□
2. Were the study subjects and the setting described in detail?	□	□	□	□
3. Was the exposure measured in a valid and reliable way?	□	□	□	□
4. Were objective, standard criteria used for measurement of the condition?	□	□	□	□
5. Were confounding factors identified?	□	□	□	□
6. Were strategies to deal with confounding factors stated?	□	□	□	□
7. Were the outcomes measured in a valid and reliable way?	□	□	□	□
8. Was appropriate statistical analysis used?	□	□	□	□

## Appendix B

**Table A2:** JBI Critical Appraisal Checklist for Qualitative Research.

	Yes	No	Unclear	Not Applicable
Is there congruity between the stated philosophical perspective and the research methodology?	□	□	□	□
2. Is there congruity between the research methodology and the research question or objectives?	□	□	□	□
3. Is there congruity between the research methodology and the methods used to collect data?	□	□	□	□
4. Is there congruity between the research methodology and the representation and analysis of data?	□	□	□	□
5. Is there congruity between the research methodology and the interpretation of results?	□	□	□	□
6. Is there a statement locating the researcher culturally or theoretically?	□	□	□	□
7. Is the influence of the researcher on the research, and vice-versa, addressed?	□	□	□	□
8. Are participants, and their voices, adequately represented?	□	□	□	□
9. Is the research ethical according to current criteria or, for recent studies, and is there evidence of ethical approval by an appropriate body?	□	□	□	□
10. Do the conclusions drawn in the research report flow from the analysis, or interpretation, of the data?	□	□	□	□
